# Unpacking Post-Traumatic Stress Disorder and Mental Health in Internally Displaced Persons: A Mediation-Moderation Model of Psychological Capital and Perceived Social Support

**DOI:** 10.3390/ijerph22121788

**Published:** 2025-11-26

**Authors:** Adane Kefale Melese

**Affiliations:** Department of Psychology, College of Education and Behavioral Sciences, Woldia University, Woldia Town P.O. Box 400, Ethiopia; adanekefale@gmail.com or adane.km@wldu.edu.et

**Keywords:** post-traumatic stress disorder, perceived social support, psychological capital, mental health, internally displaced persons

## Abstract

Internally displaced persons (IDPs) face severe physical, emotional, and social challenges due to conflict, climate change, and other crises. Ethiopia has the highest number of IDPs in Africa, primarily due to ethnic conflicts and climate-related disasters, placing them at a high risk for post-traumatic stress disorder (PTSD) and psychological distress (anxiety, emotional well-being, and depression, referred to as mental health (MH)). This study examines PTSD’s direct predictive role on IDPs’ (MH) in Debre Berhan Town, Ethiopia, the mediating role of psychological capital (PsyCap), and the moderating role of perceived social support (PSS). It also explores the interaction between PSS and PsyCap in the PTSD and MH relationship. A sample of 273 IDPs (129 females, 144 males) was selected using simple random sampling from a total population of 19,349 IDPs. Data were collected using validated instruments, including the PTSD Checklist-Civilian Version (PCL-C), PsyCap, PSS, and the General Health Questionnaire (GHQ). A structural equation modeling (SEM) analysis revealed that PTSD significantly and negatively predicts the MH of IDPs. Additionally, PsyCap positively influences their mental well-being and partially mediates the relationship between PTSD and depressive symptoms. Furthermore, PSS moderates the PTSD and MH relationship, reducing its negative impact. The finding concludes that despite PTSD directly predicting the MH of IDPs, PsyCap helps mitigate these effects. Key components of PsyCap, including hope, resilience, self-efficacy, and optimism, buffer the adverse effects of PTSD on MH. IDPs with stronger psychological resources are less likely to experience psychological distress. PSS further weakens PTSD’s negative impact, as individuals with higher PSS are less likely to suffer from trauma-related distress or depression after displacement. This study highlights the importance of PsyCap in enhancing the mental well-being of IDPs. Future research should expand on these findings and explore the integration of PsyCap-based interventions into IDP mental health programs. Strengthening social support can also provide vital support in helping IDPs cope with trauma and improve their overall psychological health.

## 1. Introduction

People are internally displaced because of natural and manmade factors [1,2,3]. Internal displacement has become one of the most pressing humanitarian challenges of the contemporary world, driven by a complex interplay of natural disasters, armed conflict, and widespread violence [1,2,4,5]. According to the United Nations High Commissioner for Refugees (UNHCR), the revised definition of internally displaced persons (IDPs) is individuals or groups of people who are compelled to leave their homes due to armed conflict, widespread violence, human rights abuses, or natural disasters. Still, they have not crossed an international border [6,7]. Unlike refugees, IDPs remain within their national territory; as a result, IDPs often face severe vulnerabilities, including inadequate protection, limited access to essential services, and restricted livelihood opportunities [8,9].

By the end of 2024, the global number of IDPs reached an unprecedented 83.4 million, an alarming increase of 7.5 million from the previous year, due to conflict and environmental disasters [10]. To contextualize the magnitude of this crisis, it is important to consider the broader historical and regional trends shaping internal displacement. The global IDP population has escalated sharply in recent years, reflecting deepening vulnerabilities and recurring displacement cycles [9]. For instance, while Africa alone accounted for 11.6 million IDPs in [11], by the end of 2024, this figure had more than tripled. In 2021, global displacement stood at 84 million, with a significant share coming from Sub-Saharan Africa [3,12]. The increment is due to both conflict-driven and disaster-induced displacements in 2024, nearly doubling the average of the past decade, signaling a growing strain on national capacities to respond effectively [10]. The protracted nature of crises, particularly in fragile contexts, such as Sudan, where 14.3 million individuals were displaced by the end of 2024, further exacerbates humanitarian needs [9].

East Africa, particularly Ethiopia, continues to contend with a complex and evolving displacement crisis, hosting both IDPs and refugees from neighboring countries, such as Sudan, South Sudan, Eritrea, and Somalia [9,13]. This regional widespread displacement is driven by multifaceted and interconnected factors, including protracted armed conflicts, climate change-induced droughts, famine, and severe food insecurity [13,14]. These challenges are further exacerbated by persistent ethnic tensions, political instability, and competition over increasingly scarce resources, creating a volatile landscape that fuels recurrent displacement [5,13]. The multi-layered nature of these drivers has made displacement a chronic humanitarian concern with profound implications for security, development, and public health in the region.

Within Ethiopia, recurrent armed conflict and political unrest have intensified internal displacement over the past three decades, particularly in the last few years [15,16]. The conflict between the federal government and regional armed groups, combined with natural disasters, has displaced over 4.5 million people as of mid-2024 [16]. Regions such as Afar, Amhara, and Tigray have been especially hard hit, while ethnic-based violence has caused significant displacement from Benishangul and Oromia into Amhara. As of early 2023, more than 580,000 IDPs remain in Amhara, many living in overcrowded and substandard conditions across various camps [17].

Beyond the immediate disruption of physical relocation, internal displacement exposes individuals to profound emotional, psychological, social, and economic detachment from their communities, heightening vulnerability to distress and long-term psychosocial distress [10,18,19]. Displaced individuals often struggle to integrate into host communities, with limited access to psychosocial support systems further exacerbating their hardship [20,21]. Among the common challenges are unmet basic needs, exposure to human rights violations, disease outbreaks, and restricted access to education and healthcare services [22]. Psychological trauma is especially prevalent among those displaced by violence, manifesting in heightened levels of anxiety, depression, social stigma, and PTSD [23,24]. PTSD poses a severe threat to individual and collective well-being, as exposure to violence and insecurity often results in persistent fear, intrusive memories, and emotional numbness across all age groups [25,26].

### 1.1. The Relationship Between PTSD and MH Among IDPs

PTSD is a severe psychiatric condition triggered by exposure to traumatic events such as war, natural disasters, or forced displacement [27,28,29]. It presents with hallmark symptoms, including intrusive memories, emotional dysregulation, avoidance behaviors, and hyperarousal (American Psychiatric Association [APA] [29,30]. PTSD often co-occurs with comorbid conditions such as anxiety and depression associated with marked impairments in daily functioning and reduced quality of life [29,31]. The prevalence of PTSD among IDPs ranges from 30% to 70%, significantly higher than in the general population [29].

PTSD is the most common mental health issue among IDPs, often co-occurring with other conditions, such as substance abuse, depression, and anxiety, which exacerbate their psychological distress [32]. A study conducted in Sudan highlights that IDP women experience higher rates of depression and severe PTSD [33]. The ongoing instability and stress of displacement also contribute to increased suicidal thoughts and self-harming behaviors [34]. Exposure to violence, including witnessing murder, sexual abuse, and devastation, further elevates the risk of PTSD [35]. Additionally, the loss of significant others and community structures, which are crucial for emotional resilience, worsens the mental health of displaced people [36]. Economic hardship, job insecurity, and limited access to mental health services also intensify stress and contribute to worsening mental health conditions [22,37]. PTSD severely impacts the daily functioning and social reintegration of IDPs, causing social isolation, emotional dysregulation, and cognitive deficits, which hinder their ability to secure stable employment [38,39]. Children and adolescents are vulnerable to long-term emotional and developmental disruptions [21]. Moreover, psychological distress, like stress, depression, or PTSD, has been linked to increased physical health issues, such as cardiovascular diseases, chronic pain, and weakened immune function [40].

Thus, PTSD not only increases the likelihood of psychiatric disorders but also significantly disrupts social and occupational functioning. The connection between PTSD and the mental health of IDPs, including depression, anxiety, and the emotional well-being of IDPs, is complex and multifactorial. Based on this body of literature, the following hypothesis was proposed: (**H1:** *PTSD significantly predicts the MH of IDPs*).

### 1.2. The Relationship Between PTSD and PSS Among IDPs

PTSD has profound psychological effects that disrupt individuals’ everyday lives [37]. It is marked by symptoms such as hypervigilance, intrusive recollections, emotional instability, and avoidance behaviors, which substantially impair functional capacity [41]. These symptoms are often more pronounced among IDPs, who are frequently exposed to persistent stressors, including substandard living environments, unemployment, and mental health-related stigma [37]. The intensity of PTSD symptoms has been shown to correlate with levels of PSS. Social support functions as a crucial protective factor, mitigating PTSD symptoms and enhancing psychological resilience [42]. It is obvious that when people are forced to leave their homes, they often lose their usual support systems, such as family, friends, and community ties [43]. This loss can lead to feelings of loneliness and long-term emotional difficulties for IDPs [44]. Therefore, examining the interplay between PTSD and PSS is critical for designing effective MH interventions tailored to the needs of displaced populations. According to [45], social support mitigates the negative effects of stressful events by lowering the risk of acquiring PTSD or easing its symptoms. To help traumatized people recover and adjust psychologically, social support is a vital protective factor that lessens the psychological effects of PTSD by fostering mental health and emotional resilience [42,46].

Social support encompasses a range of emotional, practical, and informational assistance provided by family members, close friends, and the broader community, which can buffer the adverse effects of trauma and promote long-term recovery [38,47]. Empirical research findings revealed that individuals with strong social support networks are more resilient and better equipped to cope with trauma, exhibiting lower levels of PTSD symptoms and improved psychological well-being [34,48,49]. For instance, IDPs with strong social connections reported far less PTSD-related distress than those who were socially isolated [50]. However, despite its protective benefits, many IDPs face barriers to accessing social support due to factors such as family separation, social discrimination, cultural stigma, and limited psychosocial services [36]. Thus, based on the literature review, the following hypothesis was formulated (**H2:** *PSS positively predicts MH*).

### 1.3. Moderating Role of PSS in the Relationship Between PTSD and MH

Documented evidence revealed that social support not only buffers against the effects of PTSD but also plays a vital moderating role in shaping MH outcomes among IDPs [34,48]. It fosters resilience and aids psychological recovery by providing both emotional and instrumental resources [51]. Emotional support from family and peers enhances feelings of safety and belonging, while practical assistance such as financial aid and access to healthcare alleviates trauma-related stressors [38,52]. By shaping how individuals cope with adversity, social support not only mitigates PTSD symptoms but also promotes more adaptive and sustained mental health outcomes in displaced populations [51].

Community-based support systems further enhance coping capacities and contribute to improved long-term mental health outcomes [53]. Evidence also suggests that strengthening social networks through counseling and community engagement initiatives improves psychological well-being among IDPs [35].

Research findings highlight the critical role of social support and community integration in promoting mental health and recovery in people with severe mental illnesses [54,55]. Community-based programs facilitate the integration into mental health services and foster broader social inclusion, enhancing individuals’ sense of belonging [55,56]. Social support, whether from family, peers, or mental health professionals, serves as a foundational element in recovery processes [54,57]. It has been demonstrated that focused interventions, including family-centered therapy, peer-led projects, and community engagement tactics, improve these support networks. Furthermore, PTSD symptoms can be reduced and psychological well-being enhanced by trauma-informed care models that incorporate social support systems [37]. Among IDPs, fostering hope alongside social support has also been associated with lower PTSD symptomatology, emphasizing their combined importance in mitigating displacement-related mental health challenges [51]. However, the effectiveness of social support can vary depending on the severity of trauma exposure [38]. A study among Eritrean and Sudanese male asylum seekers in Israel found that perceived social support was associated with lower PTSD symptoms only among those with low exposure to traumatic events. For individuals with high exposure, social support did not significantly mitigate PTSD symptoms, suggesting that the protective effects of social support may be limited in cases of severe trauma [58]. Thus, social support generally enhances MH (psychological distress), but its impact may vary with trauma severity. As a key moderating factor, it can buffer the psychological effects of PTSD and support recovery among displaced individuals. Based on this, the following hypothesis was proposed (**H3:** *PSS moderates the relationship between PTSD and MH*).

### 1.4. The Mediation Role of PsyCap in the Relationship Between PTSD and MH

Psychological capital (PsyCap) has become a crucial concept in the study of mental health, especially in vulnerable populations like IDPs [59]. PsyCap is a collection of positive psychological resources that improve well-being and act as a buffer against psychological distress [60]. It is characterized by four essential elements: hope, efficacy, resilience, and optimism (HERO) [60,61]. Particularly in reaction to trauma, these strengths promote psychological resilience, emotional control, and adaptive coping [61]. PsyCap is becoming more widely acknowledged as a protective element that improves mental health outcomes in high-risk situations [61]. Notably, empirical research has shown that PsyCap reduces the detrimental emotional effects of trauma, hence mediating the link between PTSD and psychological discomfort [60,62]. Furthermore, it is positively linked to subjective well-being, underscoring its role in fostering psychological adjustment among trauma-exposed populations [60].

Numerous empirical studies revealed that PsyCap plays a crucial role in fostering resilience and psychological well-being [62,63]. Moreover, research shows that PsyCap is negatively associated with psychological distress and positively linked to mental health outcomes [64]. For instance, ref. [65] indicated that psychological distress was reduced among Chinese nursing students who had higher PsyCap. Similarly, ref [64] found that PsyCap’s protective role was reinforced when well-being mediated the association between PsyCap and distress among international students.

PsyCap’s positive function in PTSD-related outcomes is being supported by empirical evidence. For instance, among trauma survivors, veterans, and medical professionals, higher PsyCap levels have been associated with fewer PTSD symptoms and improved mental health [66]. This implies that improving PsyCap could lessen distress associated with trauma. Additionally, research shows that PsyCap regulates the link between psychological discomfort and traumatic situations, such as those experienced by IDPs [58]. For instance, ref. [67] revealed that PsyCap mediated the relationship between PTSD and quality-of-life factors among Kermanshah earthquake victims. The association between psychological adjustment issues and PTSD has also been found to be mediated by psychological flexibility [68].

Additionally, empirical evidence underscores the importance of PsyCap in trauma-related mental health outcomes. ref. [66] found that PsyCap mediates the link between PTSD and other mental health conditions, with its core components promoting well-being and reducing symptoms. The study also showed that PsyCap-based interventions, such as resilience training, alleviate PTSD symptoms and buffer the impact of stress, particularly among healthcare workers [66]. Similarly, among recently hired nurses, ref. [58] found PsyCap to be a significant mediator between coping methods and mental health literacy. Additionally, ref. [69] revealed that PsyCap modulated the relationship between gratitude and COVID-19 among Chinese college students. Collectively, these studies highlight PsyCap’s critical function in enhancing coping, reducing distress, and strengthening mental health, particularly within high-risk and trauma-exposed populations. Based on these literature reviews, the following hypothesis is proposed (**H4:** *PsyCap mediates the relationship between PTSD and MH of IDPs*).

## 2. Methods

### 2.1. Participants and Setting

This study used a quantitative cross-sectional survey design, a non-experimental method that facilitates the collection of data at a single point in time to examine relationships among key variables within a defined context [70,71]. A simple random sampling technique was utilized to ensure an unbiased representation of the target population. According to the Debre Berhan Town Administration (2024), an estimated 19,349 IDPs resided in the Debre Berhan Town IDP camps. From this population, a sampling frame of 2457 individuals was identified based on eligibility criteria, including literacy, educational attainment, and age. The remaining 16,892 individuals were excluded due to being under 18, over 55, or lacking the ability to read, write, or communicate in Amharic. A random sample of 344 eligible participants was initially selected; however, 71 responses were omitted from the final dataset due to incompleteness or non-responsiveness. The final sample consisted of 273 participants (129 females and 144 males) aged between 20 and 45 years.

### 2.2. Procedures and Ethics

This study received ethical clearance from the Institutional Review Board (IRB) of Woldia University, College of Education and Behavioral Sciences, through the Research and Community Services Coordinating Office (Approval No. RCSO 193/2024). Furthermore, official permission was obtained from the Debre Berhan town municipality, authorizing the recruitment of participants and the collection of data from IDPs residing in the designated camps within Debre Berhan town. To ensure linguistic and cultural accuracy, all data collection instruments were first translated from English to Amharic by a professional translator and then back-translated into English by a different translator. The two English versions were compared, and any discrepancies were resolved through consensus. A pilot test was conducted with 50 participants to evaluate the clarity and relevance of the instruments. Before data collection, the participants were informed about the purpose of the study, confidentiality measures, and their rights. Oral informed consent was obtained from each participant. Two trained data collectors with academic backgrounds in psychology facilitated the process. Following ethical clearance, data collection was carried out from September to November 2024.

### 2.3. Instrumentation

Regarding instrumentation, a self-designed questionnaire was utilized to collect demographic information, including age, gender, religion, educational level, and place of displacement. In addition, data on the study’s primary variables were obtained using the following standardized measurement tools:

### 2.4. PTSD (PCL-C)

The PTSD Checklist Civilian Version (PCL-C), developed by [72] at the National Center for PTSD, is a widely used self-report instrument designed to assess PTSD-traumatic stress disorder symptoms in civilian populations based on the DSM-IV diagnostic criteria. Adapted from the military version (PCL-M), the PCL-C evaluates PTSD symptoms related to a broad range of traumatic experiences, not limited to combat exposure. The scale comprises 17 items, each rated on a five-point Likert scale ranging from 1 (Not at all) to 5 (Extremely), reflecting symptom severity over the past month. These items are categorized into three core symptom clusters: re-experiencing (5 items), avoidance/numbing (7 items), and hyperarousal (5 items). The total score ranges from 17 to 85, with higher scores indicating more severe PTSD symptoms. A score between 30 and 35 is typically used as a screening threshold, while scores of 44 or above may indicate a clinical level of PTSD. The PCL-C exhibits excellent internal consistency, with Cronbach’s alpha values ranging from 0.94 to 0.97 and strong construct validity through high correlations with the Clinician-Administered PTSD Scale (CAPS). Its strengths include direct alignment with the DSM-IV criteria, the ability to assess both symptom presence and severity, and adaptability to different types of traumas. Furthermore, the instrument has been translated into several languages to enhance its applicability in diverse clinical and research contexts.

### 2.5. Psychological Capital Scale

The Psychological Capital Questionnaire (PCQ-24), developed by [60], measures PsyCap, which includes hope, self-efficacy, resilience, and optimism (HERO). The PCQ-12, a shortened version, contains 12 items across 4 subscales: hope (4 items), self-efficacy (3 items), resilience (3 items), and optimism (2 items), with responses rated on a six-point Likert scale (1 = Strongly Disagree to 6 = Strongly Agree). This scale provides an overall PsyCap score and individual dimension scores. The PCQ-12 has been validated in various cultural settings, including China [60] and Spain [73]. It demonstrates strong internal consistency, with Cronbach’s alpha ranging from 0.70 to 0.90. Due to its brevity and strong psychometric reliability, the PCQ-12 is widely used in organizational, educational, and psychological research to assess positive psychological resources.

### 2.6. General Health Questionnaire

The General Health Questionnaire (GHQ), originally developed by [74], is a widely recognized instrument for evaluating psychological distress and overall mental health. The GHQ-12, a concise 12-item version introduced by [74], is specifically designed to detect psychological distress, making it particularly suitable for use in non-clinical populations. While its primary purpose is to screen for the risk of mental disorders, it is also employed to assess general symptom burden and mild psychological difficulties. The GHQ-12 evaluates symptoms related to anxiety, social functioning, and somatic concerns using a four-point Likert scale (ranging from 0 = “Not at all” to 3 = “More than usual”). Its high internal consistency, with Cronbach’s alpha values reported between 0.73 and 0.91, demonstrates its reliability as a tool for assessing mental health in community-based and general population studies [75].

### 2.7. The Interpersonal Support Evaluation List (ISEL)

The Interpersonal Support Evaluation List (ISEL), developed by [76], measures perceived social support. A shortened 12-item version (ISEL-12) was later introduced to reduce respondent burden while maintaining the core elements of the original scale of 40 items [77,78]. The ISEL-12 assesses three dimensions: Appraisal Support (guidance), Belonging Support (companionship), and Tangible Support (practical help), with four items per subscale rated on a four-point Likert scale (1 = Definitely False to 4 = Definitely True). It provides both total and subscale scores. The ISEL-12 has demonstrated good reliability (α = 0.70–0.90) and is widely used across various cultural and clinical and non-clinical study contexts.

### 2.8. Data Analysis

The collected data were systematically coded, organized, and entered into a statistical package for social sciences (SPSS) version 28.0 for validation and error detection. Descriptive statistics (mean and standard deviation) and correlational analyses were conducted. Structural equation modeling (SEM) was carried out using AMOS version 28.0. Before SEM, a confirmatory factor analysis (CFA) was performed to assess the validity of the measurement model, utilizing the maximum likelihood (ML) estimation method [79]. The model fit was evaluated using several indices, including chi-square (χ^2^), standardized root mean square residual (SRMR), root mean square error of approximation (RMSEA), comparative fit index (CFI), and Tucker–Lewis index (TLI). A mediation analysis was conducted using PROCESS Macro version 4.2 (Model 1), which estimated three regression pathways.

## 3. Results

### 3.1. Descriptive Statistics

Table 1 displays the descriptive statistics and correlations among the study variables. The participants reported moderate PTSD symptoms (M = 56.79, SD = 13.73) and psychological distress (MH; M = 19.70, SD = 7.56), exceeding the typical clinical cut-offs. The PsyCap levels were relatively high (M = 47.21, SD = 11.76), while PSS was low (M = 13.09, SD = 6.87), indicating limited social support. Skewness and kurtosis values (−1 to +1) confirmed normal distribution for all variables, supporting the use of parametric tests. PTSD was positively correlated with MH (*r* = 0.517, *p* < 0.01) and negatively correlated with PsyCap (*r* = –0.460, *p* < 0.01) and PSS (*r* = –0.402, *p* < 0.01). MH also negatively correlated with PsyCap (*r* = –0.409, *p* < 0.01) and PSS (*r* = –0.528, *p* < 0.01). A strong positive correlation emerged between PsyCap and PSS (*r* = 0.556, *p* < 0.01), suggesting that psychological and social resources are interrelated, while higher distress is linked to lower support and resilience.

### 3.2. Construct Reliability and Validity Measures

Table 2 presents the reliability and validity results for the study constructs. All standardized loadings exceeded 0.70, and the average variance extracted (AVE) values ranged from 0.521 to 0.587, confirming acceptable convergent validity. The construct reliability (CR) values were above 0.70, indicating strong internal consistency [80]. Discriminant validity was supported using the Fornell–Larcker criterion: the square root of each AVE exceeded the inter-construct correlations, and the AVE values were greater than the corresponding maximum shared variance (MSV) values. For instance, PsyCap’s AVE (0.587) and √AVE (0.766) exceeded its MSV (0.309) and correlation with PSS (*r* = 0.556), confirming the distinctiveness of each variable. Below is a summary of the key findings for the variables PTSD, HM, PsyCap, and PSS:

### 3.3. Measurement Model Fit Analysis of the Variables

SEM was employed to assess the hypothesized relationships among PTSD, HM, PsyCap, and PSS. The model exhibited an acceptable fit to the observed data. To evaluate the overall adequacy of the measurement model, a range of goodness-of-fit indices was examined. As summarized in Table 3, CFA provides satisfactory model fit statistics, with all indices meeting or exceeding the recommended thresholds (χ^2^/df = 1.34, *p* < 0.05, CFI = 0.949, TLI = 0.958, SRMR = 0.053, RMSEA = 0.041). These results provide empirical support for the adequacy of the measurement model, thereby justifying its use in subsequent structural analyses to test the proposed research hypotheses.

### 3.4. Hypothesis Tests for the Proposed Structural Model

**Figure 1:**
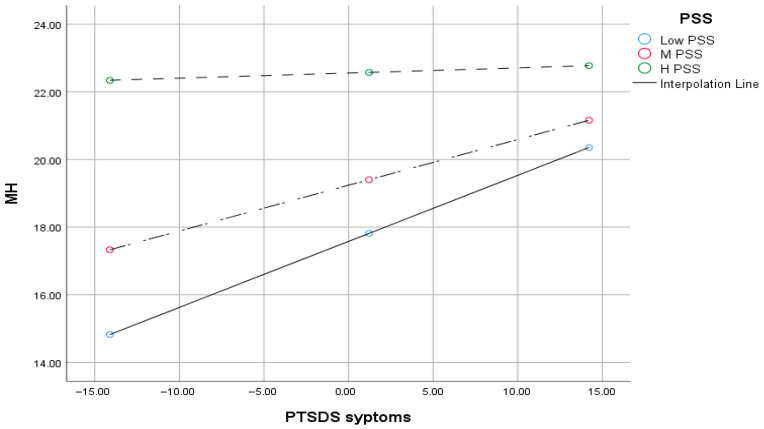
Visual representation of the moderation role of PSS in PTSD and MH relationships. The different dotted lines illustrate the moderation effect by showing the simple slopes of PTSD symptoms on mental health at low, medium, and high levels of perceived social support (PSS). MH = Mental health; PSS = Perceived Social Support; PTSDS = Post-traumatic Stress Disorder Symptoms.

Following the validation of the measurement model, SEM was utilized to test the hypothesized relationships among the latent constructs PTSD, MH, PsyCap, and PSS (see Figure 1). The structural model demonstrated an acceptable fit based on standardized path coefficients, significance levels, and fit indices, supporting the examination of causal mediation effects among the latent variables.

### 3.5. Structural Model Fit Assessment

As presented in Table 4, the structural model results support the hypothesized relationships among PTSD, MH, PsyCap, and PSS. PTSD significantly predicted poorer mental health (β = 0.233, *p* < 0.001) and lower PsyCap (β = 0.673, *p* = 0.026). PsyCap was strongly associated with improved mental health (β = 0.720, *p* < 0.001) and significantly mediated the PTSD and MH relationship (β = 0.564, *p* < 0.001). PTSD also significantly predicted PSS (β = 0.422, *p* = 0.034), which negatively influenced PsyCap (β = 0.380, *p* = 0.038, CI [0.230, 0.523]). Additionally, PSS significantly mediated the PTSD and PsyCap relationship (β = 0.324, *p* = 0.011, CI [0.133, 0.310]). Overall, the structural model findings support the proposed pathways and highlight the mediating roles of PsyCap and PSS.

### 3.6. Moderation Effect of PSS in the Relationship Between PTSD and MH

As indicated in Table 5, a moderation analysis was conducted using the PROCESS macro version 4.2 (Model 1; ref. [82]) to investigate whether PSS moderates the relationship between PTSD symptoms and MH outcomes among IDP study participants. The overall model was statistically significant, F (3, 269) = 20.83, *p* < 0.001, with an R^2^ of 0.189, indicating that approximately 18.9% of the variance in MH was accounted for by PTSD symptoms, PSS, and their interaction. These findings suggest that PSS plays a moderating role in the association between PTSD and MH outcomes in this population.

### 3.7. Conditional Effects of PTSD on MH at Levels of PSS

As shown in Table 6, At low and moderate levels of PSS, PTSD symptoms significantly predicted poorer MH. However, at high levels of PSS, the relationship was not significant, indicating a buffering effect of social support.

## 4. Discussion

The main objective of this study was to examine the predictive effects of PTSD on MH with PsyCap as a mediator and PSS as a moderator role among IDPs. The first hypothesis confirmed a negative association between PTSD and mental health. Findings indicate both direct and indirect impacts of PTSD on the psychological well-being of IDPs, aligning with prior research. IDPs frequently endure traumatizing events that seriously impair their psychological health, such as abuse, assault, and deprivation of basic needs services [83,84]. The risk of mental health conditions, including PTSD, anxiety, and depression, is significantly increased by the combined effects of these hardships, especially when combined with continuous displacement brought on by conflict and instability [85,86].

Additionally, psychosocial issues, like social isolation, unemployment, and the disintegration of community networks, increase their susceptibility, which often shows up as PTSD symptoms like emotional detachment, intrusive memories, and hypervigilance [87,88,89]. These intersecting stressors underscore the urgent need for comprehensive mental health interventions tailored to displaced populations. In line with the current study, furthermore, empirical evidence indicated that mental well-being issues such as PTSD, depression, anxiety, and physical complaints are widely reported among IDPs [13,32,88,90]. These conditions often stem from traumatic experiences, lack of social support, and restricted access to care, leading to lasting psychological harm, including suicidal ideation [90]. IDPs frequently endure severe hardships like torture, family separation, and loss of loved ones [91]. Moreover, studies show that displaced individuals are more likely to develop mental disorders than non-displaced populations [50], with similar patterns observed in conflict-affected areas such as Uganda and Ukraine [92].

The second hypothesis proposed that PsyCap mediates the relationship between PTSD and MH. The findings supported this, showing that PsyCap partially mediates the impact of PTSD on the mental well-being of IDPs. This suggests that PsyCap, which consists of the positive psychological resources of optimism, resilience, self-efficacy, and hope, is essential for improving MH outcomes, especially for people who have experienced trauma. Research indicates that PsyCap not only promotes psychological well-being but also lessens depressive symptoms by encouraging optimistic thinking and adaptive coping strategies [93]. Additionally, PsyCap buffers the negative consequences of trauma and promotes psychological adjustment, acting as a crucial mediating component in the link between PTSD and MH [93]. In line with the current study, prior empirical research findings revealed that PsyCap acts as a protective buffer that improves psychological health and resilience while also partially mediating the association between PTSD and IDPs’ MH [62,93]. Reduced symptoms of depression and anxiety are consistently associated with higher-order PsyCap levels, especially in trauma-affected groups like veterans and displaced people [94].

In line with the findings of the current study, ref. [71] indicated that PsyCap, which consists of hope, resilience, optimism, and self-efficacy, seems to improve psychological well-being and lessen depressive symptoms in populations afflicted by trauma. Consistent with previous research, higher levels of PsyCap were associated with lower levels of anxiety and depression, particularly among vulnerable groups such as IDPs and veterans [68]. This study supports the mediating role of PsyCap in the PTSD and MH relationship [67,95]. PsyCap’s ability to mitigate the effects of stress and promote trauma recovery is further supported by meta-analytic and intervention-based research [66].

Despite the hardships of displacement, many IDPs display psychological strength supported by their internal resources. PsyCap fosters effective coping and recovery by enhancing emotional regulation and stress management [13,93]. Individuals with strong PsyCap are often better equipped to process trauma and maintain psychological balance [96,97]. Resilience plays a vital role for those with higher resilience to adapt more effectively to adversity, while those with lower resilience face greater emotional challenges [98].

Hypothesis three tested whether PSS moderates the relationship between PTSD and MH among IDPs. The hypothesis accepted that PSS is the moderator in the relationship between PTSD and MH. The findings of this study confirmed the moderating role of PSS, underscoring its function as a crucial protective factor that buffers the adverse psychological impact of PTSD on mental well-being. This finding is consistent with a substantial body of literature demonstrating that social support can alleviate psychological distress and foster resilience, particularly among trauma-affected populations [99]. It has been demonstrated that social support is particularly important for improving adaptive coping and preventing MH decline among displaced populations [100]. Additionally, there is empirical evidence that PSS moderates the relationship between PTSD symptoms and MH outcomes, including overall quality of life, psychological distress, and suicidal thoughts [101,102]. In essence, higher levels of PSS buffer the negative impact of PTSD on MH, highlighting its protective role in trauma recovery.

This study shows that PSS can mitigate the detrimental effects of PTSD symptoms on MH, hence supporting its moderating role [103]. By improving emotional resilience, PSS seems to protect people against trauma-related psychological decline, which is in line with the buffering theory [77]. Even in the face of severe trauma, people with good support systems show fewer signs of distress and a decreased risk of suicidal thoughts [102,104]. Social support in displacement contexts not only lessens symptoms of depression and PTSD [105,106] but also promotes resilience and improved coping mechanisms, which, in turn, improve physical health by lowering physiological strain caused by stress [43]. Furthermore, evidence shows that PSS can moderate PTSD and MH relationships, though its effectiveness varies by symptom type and context. While support often reduces distress, its buffering effect may be limited among displaced populations without broader systemic resources [44]. Nonetheless, in refugee settings, social support and resilience assist psychological adjustment, emphasizing their value in trauma-informed interventions [107].

The finding confirmed the fourth hypothesis, which postulated that PSS mediates the association between PsyCap and PTSD. This suggests that PSS is essential for reducing PTSD symptoms and helping IDPs develop psychological resources, including hope, resilience, and self-efficacy [43,105]. In line with earlier studies, PSS has been demonstrated to act as a mediating mechanism in a variety of settings, connecting psychological strengths and trauma exposure [108], underscoring its importance in fostering psychological recovery and adaptive functioning. In the intricate relationship between psychological resources, MH, and trauma, PSS acts as a mediator as well as a buffer [53]. PSS promotes the development of PsyCap, a construct that consists of hope, efficacy, resilience, and optimism, by improving the coping ability [60]. In turn, PsyCap has been demonstrated to adversely predict psychological distress and PTSD [93]. This is especially important for IDPs who suffer from compounded trauma and frequently have interrupted support networks [109].

Additionally, there is growing empirical evidence that PSS improves PsyCap, which mitigates the psychological impacts of PTSD [62]. PsyCap, which includes hope, resilience, optimism, and self-efficacy, has been demonstrated to be strengthened by high levels of social support, reducing the negative effects of stress and trauma [61]. These psychological tools are vital for coping with IDPs, and PSS is crucial to their growth [43,97]. Furthermore, PSS mediates the link between resilience and social support, highlighting the importance of people’s support perceptions for psychological adjustment [110]. These findings underscore the importance of integrated interventions that simultaneously strengthen social support networks and PsyCap to reduce PTSD symptoms and enhance mental health in displacement contexts [107].

## 5. Summary, Conclusions, and Recommendations

This study examined the predictive effects of PTSD on MH among IDPs, with PsyCap as a mediator and PSS as a moderator. The findings revealed that PTSD significantly affects MH both directly and indirectly. Traumatic experiences, such as violence and forced displacement, were found to impair psychological well-being. PsyCap partially mediated the relationship between PTSD and MH by enhancing individuals’ resilience, hope, and self-efficacy, which contributed to reduced psychological distress. Moreover, PSS moderated this relationship, acting as a protective factor that buffers the negative effects of trauma. These findings highlight the critical role of PsyCap and PSS in promoting psychological recovery among IDPs. Strengthening these resources through targeted interventions may improve the mental health outcomes of trauma-affected populations. Therefore, strategies aimed at enhancing internal coping resources (PsyCap) and external support systems (PSS) are essential in addressing the psychological needs of displaced individuals.

Despite its contributions, the study is not without limitations. One key limitation is the approximately 20% non-response rate (71 out of 344), which may affect the generalizability of the findings. This level of non-participation could introduce bias if the experiences or mental health conditions of the non-respondents differ meaningfully from the ones of those who participated. Future research should aim to increase the response rates and consider mixed-method approaches to capture a broader range of experiences among IDPs. Based on the findings, the following recommendations are proposed: In addition to this, this study may be subject to common method bias due to its reliance on self-report measures. Furthermore, sampling from selected IDP camps may limit the generalizability of the findings.

To the academic community: Future studies should investigate other psychological variables such as depression, anxiety, and the individual components of PsyCap concerning PTSD and MH outcomes among displaced populations.

To governmental and non-governmental institutions: Coordinated efforts among governments, NGOs, and religious institutions are necessary to provide integrated support—including financial aid, material assistance, education, and employment opportunities—to improve the living conditions of IDPs and support their return to normalcy.

To physical and psychological health professionals: Mental health programs should incorporate approaches that build PsyCap, foster social support, and offer professional psychological services. Training and interventions should be designed to integrate these factors to enhance mental well-being and resilience among IDPs.

## Figures and Tables

**Table 1:** Descriptive statistics and Pearson’s correlation matrix among the variables.

Variable	Min	Max	Mean	SD	Skewness	Kurtosis	1	2	3	4
**PTSD**	24.00	116.00	56.79	13.73	0.08	0.12	—	0.517 **	−0.460 **	−0.402 **
**HM**	6.00	36.00	19.70	7.56	0.27	−0.99	—	—	−0.409 **	−0.528 **
**PsyCap**	17.00	72.00	47.21	11.76	−0.41	−0.31	—	—	—	0.556 **
**PSS**	3.00	33.00	13.09	6.87	0.63	−0.45	—	—	—	—

** *p* < 0.001. (2-tailed): PTSD = post-traumatic stress disorder; MH = mental health; PsyCap = psychological capital; PSS = perceived social support; SD = standard deviation.

**Table 2:** Construct reliability and validity measures.

Variables	CR	AVE	MSV	PTSD	MH	PsyCap	PSS
**PTSD**	0.931	0.576	0.309	0.759			
**MH**	0.924	0.551	0.309	0.517 **	0.742		
**PsyCap**	0.934	0.587	0.309	−0.460 ***	−0.409 **	0.766	
**PSS**	0.915	0.521	0.309	−0.402 **	0.528 **	0.556 **	0.722

CR = construct reliability; AVE = average variance extracted; MSV = maximum shared variance; and the square root of the AVE in the diagonal; ** *p* < 0.01; *** *p* < 0.001.

**Table 3:** Results of the measurement model fit.

Model	*x*^2^/df	CFI	TLI	SRMR	RMSEA
**Model 1**	1.34 *	0.949	0.958	0.053	0.041

* indicates *p* < 0.05. All within the acceptable thresholds [81].

**Table 4:** Results of the structural model.

Hypothesis	Path	β	SE	*p*	CI = 95%
Hypothesis 1	PTSD → MH	0.233	0.070	***	--
Hypothesis 2	PTSD → PsyCap	0.673	0.0817	0.026	--
Hypothesis 3	PsyCap → MH	0.720	0.088	***	--
Hypothesis 4	PTSD → PsyCap→ MH	0.564	0.142	***	--
Hypothesis 5	PTSD → PSS	0.422	0.071	0.034	--
Hypothesis 6	PSS → PsyCap	0.380	0.050	0.038	CI [0.230, 0.523]
Hypothesis 7	PTSD → PSS → PsyCap	0.324	0.066	0.011	CI [0.133, 0.310]

*** *p* < 0.001.

**Table 5:** Moderation effect of PSS in the relationship between PTSD and MH interactions.

Predictor	B	SE	t	*p*	95% CI
Constant	19.931	0.427	46.719	0.000	[19.091, 20.770]
PTSD	0.110	0.034	3.198	0.002	[0.042, 0.178]
PSS	0.332	0.063	5.264	0.000	[0.208, 0.456]
PTSD × PSS	−0.012	0.005	−2.231	0.027	[−0.023, −0.001]

The interaction term was statistically significant (*p* = 0.027), indicating that PSS significantly moderates the relationship between PTSD and MH.

**Table 6:** Conditional effect of the moderation variable.

PSS Level	Effect	SE	t	*p*	95% CI
**Low (−1 SD)**	0.195	0.038	5.082	0.000	[0.120, 0.271]
**Medium (Mean)**	0.135	0.031	4.343	0.000	[0.074, 0.197]
**High (+1 SD)**	0.015	0.066	0.232	0.817	[−0.114, 0.144]

## Data Availability

The data supporting this study are not publicly available due to ethical and privacy considerations; however, they may be provided by the corresponding author upon reasonable request and with appropriate institutional approval.
